# AMPK Activators Suppress Cervical Cancer Cell Growth through Inhibition of DVL3 Mediated Wnt/β-Catenin Signaling Activity

**DOI:** 10.1371/journal.pone.0053597

**Published:** 2013-01-02

**Authors:** H. T. Kwan, David W. Chan, Patty C. H. Cai, Celia S. L. Mak, Mingo M. H. Yung, Thomas H. Y. Leung, Oscar G. W. Wong, Annie N. Y. Cheung, Hextan Y. S. Ngan

**Affiliations:** 1 Departments of Obstetrics and Gynaecology, LKS Faculty of Medicine, The University of Hong Kong, Hong Kong SAR, P. R. China; 2 Department of Pathology, LKS Faculty of Medicine, The University of Hong Kong, Hong Kong SAR, P. R. China; National Cancer Center, Japan

## Abstract

Recent evidence has suggested that AMPK activators may be applied as therapeutic drugs in suppressing cancer cell growth. However, the molecular mechanism of their suppressive function in cancer cells is still unclear. Here we show that AMPK activators impair cervical cancer cell growth through the reduction of DVL3, a positive regulator in Wnt/β-catenin signaling and an oncogenic player in cervical cancer tumorigenesis. By western blot and immunohistochemical analyses, we demonstrated that DVL3 was frequently upregulated and significantly associated with elevated β-catenin (*P* = 0.009) and CyclinD1 (*P* = 0.009) expressions in cervical cancer. Enforced expression of DVL3 elevated β-catenin and augmented cervical cancer cell growth, verifying that DVL3-mediated Wnt/β-catenin activation is involved in cervical cancer oncogenesis. On the other aspect, we noted that the cervical cancer cell growth was remarkably suppressed by AMPK activators and such cell growth inhibition was in concomitant with the reduction of DVL3 protein level in dose- and time-dependent manners. Besides, impaired mTOR signaling activity also reduced DVL3 expression. In contrast, co-treatment with Compound C (AMPK inhibitor) could significantly abrogate metformin induced DVL3 reduction. In addition, co-treatment with AM114 or MG132 (proteosomal inhibitors) could partially restore DVL3 expression under the treatment of metformin. Further *in vivo* ubiquitination assay revealed that metformin could reduce DVL3 by ubiquitin/proteasomal degradation. To our knowledge, this is the first report showing the probable molecular mechanisms of that the AMPK activators suppress cervical cancer cell growth by impairing DVL3 protein synthesis via AMPK/mTOR signaling and/or partially promoting the proteasomal degradation of DVL3.

## Introduction

Cervical cancer is one of the most common gynecological cancers worldwide. To date, the most well-known cause of cervical cancer is the human papillomavirus (HPV) infection. Persistent HPV infection promotes the development of precancerous lesions to cervical cancer [1]. However, HPV infection alone is not sufficient to transform epithelial host cells to cancer cells. Thus, other factors, such as the up-regulation of oncogenes and aberrant activation of related signaling pathways, may be involved in cervical carcinogenesis [2]. Recent evidences have shown that aberrant activation of Wnt/β-catenin signaling is crucially involved in cancer development [3], [4], [5]. The accumulation of β-catenin is the hallmark in causing aberrant activation of Wnt/β-catenin signaling which is closely associated with the carcinogenesis of uterine cervix [6], [7], [8], [9], and particularly in invasive cervical carcinomas [10]. Thus, targeting this pathway may be a promising approach in molecular therapy of this disease.

AMP-activated protein kinase (AMPK) is a master regulator of cellular energy system [11]. Activated AMPK activity impairs mechanistic target of Rapamycin (mTOR) signaling and its corresponding p70S6 kinase and 4E-BP1 activity [12], thereby inhibiting protein synthesis [13], [14] and cell growth. Therefore, it is not surprising that low AMPK activity favors carcinogenesis [15], [16]. To this end, numerous pharmaceutical AMPK activators such as A23187, A769662, 5-aminoimidazole-4-carboxamideribonucleoside (AICAR) and metformin have recently been shown to exert anti-tumor effects in numerous human cancers [17], [18], [19], [20], [21], [22], [23]. Particularly, metformin has been used in several ongoing clinical trials [24], [25], [26], [27]. However, the molecular mechanisms for the anti-tumor effects of these AMPK activators have not yet been clearly elucidated.

In this study, we showed that DVL3 was significantly upregulated and was correlated with Wnt/β-catenin activity in cervical cancer. More importantly, we are the first to provide evidence that AMPK activators inhibit cervical cancer cell growth through impairing DVL3-mediated Wnt/β-catenin signaling in cervical cancer cells. We showed that the inhibition of AMPK/mTOR-mediated protein synthesis and the enhancement of proteasomal degradation were the molecular mechanisms in the reduction of DVL3 exhibited by AMPK activators. Our findings implicate the importance of DVL3 in the cervical cancer oncogenesis and highlight the therapeutic value of targeting DVL3 by AMPK activators in cervical cancer treatment.

## Materials and Methods

### Cell culture

Five cervical cancer cell lines (HeLa, SiHa, C33A, CaSki and C41) (American Type Culture Collection, Rockville, MD) (cell lines authentication was done by in-house STR DNA profiling analysis) and two immortalized cervical epithelial cell lines (NC104 and NC105) [6] (gift from Prof. George. Tsao, Department of Anatomy, The University of Hong Kong) were used in this study. All cell lines were grown at 37°C in 5% CO2 in minimum essential medium or Dulbecco's modified Eagle medium (Gibco-BRL, Gaithersburg) with 10% fetal bovine serum (Gibco) and 1% Penicillin-Streptomycin (Gibco).

### Plasmids and cell transfection

The GFP-tagged-DVL3 expressing construct was used for GFP/DVL3 protein expression [28], while the pEGFP-C1 plasmid was used as negative control. The pCMV2-Flag/DVL3 was used for TOP/FOP luciferase reporter assay, while both pSuper8XTOPFlash and pSuper8XFOPFlash constructs were kindly provided by Dr. R. Moon (University of Washington, Washington, USA). LipofectAMINE^TM^ 200 (Invitrogen Life Technologies, Carlsbad, CA) was used for cell transfection according to the manufacturer's protocols. For the establishment of GFP-DVL3 stably expressing cells, transfected cells were selected with G418 for 2 weeks and verified by western blot analysis.

### RNA extraction and Quantitative reverse transcriptase–PCR

Total RNA was extracted by TRIzol reagent (Invitrogen) according to the manufacturer's instruction. Complementary DNA was synthesized using reverse transcription reagent kit (Applied Biosystems, Foster City). The expression of *DVL3* was evaluated by quantitative reverse transcriptase-PCR (q-PCR) in an ABI PRISM™ 7500 system (Applied Biosystems) using Taqman® Gene expression Assays; human *DVL3* (Assay ID: Hs00610263_m1). The human *18S rRNA* (Assay ID: Hs99999901_m1) was used as an internal control.

### Protein Extraction, Western blotting and Immunohistochemical (IHC) analyses

Protein lysate was extracted by lysing cells pellet with 1x lysis buffer (10X lysis buffer (Cell Signaling Technology, Danvers, MA, USA), 1∶100 PMSF, protease inhibitor and distilled water). For western blot analysis, samples containing a fixed amount of protein were separated by SDS-PAGE and electroblotted to the Hybond-P membranes (Amersham Pharmacia Biotech, Cleveland, OH, USA). Membranes were then blotted with 5% skimmed milk for 30 minutes and incubated with primary antibodies; anti-pp70S6kinase, anti-p70S6 kinase, anti-pAMPKα, anti-AMPKα, anti-CyclinD1 (Cell signaling Technology), anti-DVL1, anti-DVL2, anti-DVL3, anti-GFP (Santa Cruz Biotechnology, Santa Cruz, CA, USA) and anti-β-catenin (BD Biosciences, San Jose, CA, USA) overnight at 4°C. Anti-mouse or anti-rabbit secondary antibody conjugated to horse radish peroxide (Amersham Pharmacia Biotech, OH) was used and visualized by enhanced chemiluminescence (Amersham). For immunohistochemical analysis, cervical cancer tissue array (CXC1021) (5 cases of normal cervix/cervicitis and 97 cases cervical cancers) (Pantomics Inc, CA, USA) were used for immunohistochemical staining for DVL3, β-catenin and CyclinD1. The sections were immunostained with primary anti-DVL3(1∶200 dilution)(NB110-59941, Novus Biologicals, Littleton, CA USA), anti-β-catenin (BD Biosciences)(1∶200 dilution) and anti-CyclinD1(1∶100 dilution)(H-295, Santa Cruz Biotehcnology). Incubation with Tris-buffered saline was used as negative controls. Their expression levels were scored manually by multiplying the percentage of the proportion of immunopositive staining area (0–100%) and the intensity of staining (+1, weak; +2, moderate; +3, intense; and +4, very intense). The fold change of each gene was calculated by dividing its expression level of each cancer sample by the mean value of its expression level of normal cervix/cervicitis. The cutoff point (number of folds) of each gene was then determined by its expression levels and statistical significance. All tissue section were examined and scored independently by two investigators.

### Luciferase Reporter Assay

HEK293 cells were transiently transfected with 0, 50, 100, and 200 ng of pCMV2-Flag/DVL3 as well as either a pSuper8XTOPFlash or pSuper8XFOPFlash luciferase reporter construct. All transfections were normalized with pcDNA3.1 (+) vector. The luciferase activity was determined using the Dual-Luciferase Reporter Assay System (Promega). Transfection efficiencies were normalized with *Renilla* luciferase activity. All experiments were performed in triplicate and in 3 independent experiments.

### *In Vivo* Ubiquitination Assay

The procedure was described as previously [29]. Briefly, C33A or Hela cells were seeded onto 100 mm culture plates. The pcDNA-HA(Ub)_8_ was transient transfected into the above cell types respectively using Lipofeactamin 2000 transfection kit (Invitrogen). The cell lysate was harvested by using NET lysis buffer (50 mM Tris, pH 7.4, 150 mM NaCl and 5 mM EDTA) with 1% NP40, pH 8.0, 0.1 mM PMSF, and 1 mM Complete TM protease inhibitor cocktail (Roche)) on the cell pellet. One mg of cell lysate was incubated with 1 µg of mouse anti-IgG and anti-DVL3 (Santa Cruz) at 4°C. After incubation, 40 µl of Protein A/G Plus-Agarose beads (Santa Cruz) were added to each sample and mixed by rotating overnight at 4°C. After centrifugation, the beads were washed four times with NET lysis buffer, followed by addition of sample buffer containing DTT and boiled for 10 min prior to electrophoresis. The proteasome inhibitors, MG132 and AM114, were obtained from Calbiochem (La Jolla, CA).

### Cell viability assay

Cell proliferation kit (XTT)(Roche) was used to measure cell viability for 5 days according to the manufacturer's protocol. Three independent experiments were performed in triplicate.

### Clonogenic assay

Cells were allowed to grow for 2 weeks. After 2 weeks, medium was removed followed by incubation with methanol for 30 minutes and then stained with crystal violet for 1 hour. After the staining, cells were washed with PBS. Triplication of each sample and three independent experiments were performed and the number of cells was counted by the Gel-Doc system.

### Data analysis

Student's t test (for parametric data) and the Mann-Whitney test (for non-parametric data) were used for analysis. The clinicopathological analysis between the expression of DVL3, β-catenin, CyclinD1 and clinical parameters was analyzed by Crosstabs and Pearson Chi-Square test using the SPSS 13.0 software (SPSS, Chicago, IL). All data were expressed as mean ± SD. A *P*-value of less than 0.05 was considered as significant.

## Results

### DVL3 is frequently upregulated in cervical cancer cells

**Figure 1:**
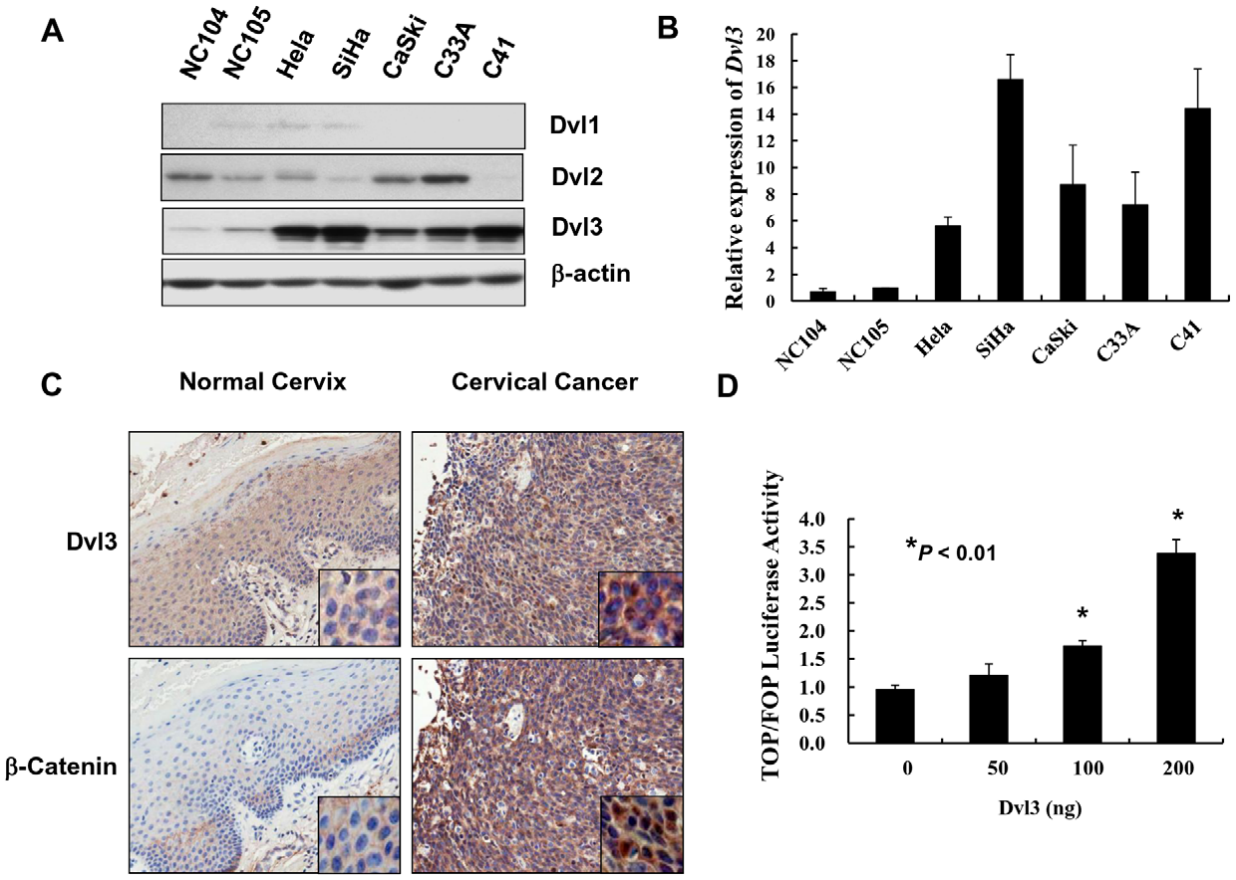
DVL3 and β-catenin are frequently up-regulated and associated with augmented Wnt/β-catenin signaling activity in cervical cancer. (A) Western blot analysis showed that only the level of DVL3 but not DVL1 and DVL2 was significantly up-regulated in cervical cancer cell lines when compared with the normal cervix cell lines. (B) Real time q-PCR revealed the mRNA of *DVL3* was obviously higher among cervical cancer cell lines when compared with the normal cervix cell lines. The value of NC105 was used to normalize other cell lines. (C) Immunohistochemical analysis showed that both DVL3 and β-catenin were upregulated on cervical cancer tissues when compared with normal cervix samples. DVL3 was distributed in the cytoplasmic region whereas β-catenin was located in both the cytoplasmic and nuclear regions. (Magnification: 20x) (D) Luciferase reporter assay revealed that transient transfection of DVL3-expressing plasmid could increase β-catenin transcriptional activity in HEK293 cells.

Emerging evidences have shown that DVLs are able to up-regulate β-catenin and promote cell growth in colorectal cancer [30], malignant pleural mesothelioma [31] and non-small-cell lung cancer [32] etc. We thus examined the expression pattern of DVLs (DVL1, DVL2 and DVL3) in cervical cancer cell lines by western blot analysis. Our data demonstrated that DVL3 but neither DVL1 nor DVL2 was significantly up-regulated in a panel of cervical cancer cell lines (Hela, SiHa, CaSki, C33A and C41) when compared with immortalized cervix cell lines (NC104 and NC 105) (Figure 1A), suggesting that DVL3 is dominantly upregulated in cervical cancer cells. We next examined the mRNA level of *DVL3* in these cell lines by real time quantitative reverse transcription PCR (q-PCR). Similarly, the *DVL3* mRNA levels were significantly higher in cervical cancer cell lines as compared with the normal immortalized cervix cell lines (Figure 1B). Collectively, these findings indicate that DVL3 is the major isoform of DVLs frequently up-regulated in cervical cancers cells.

### Overexpression of DVL3 and β-catenin in cervical cancer

To study the significance of DVL3 and β-catenin in cervical cancer, we examined the expressions of DVL3 and β-catenin on a cervical cancer tissue array (CX1021) using immunohistochemical (IHC) analysis. The immunoreactivity of DVL3 was only observed in the cytoplasm whereas β-catenin localized in both nuclei and cytoplasmic regions of both normal and cervical cancer cells (Figure 1C). IHC analysis showed that remarkable increased expressions of DVL3 and β-catenin were observed in cervical tumor sections as compared with the normal cervix sections (Tables 1 and 2). Statistical analysis showed that high expression of DVL3 (rank>5 folds) was significantly correlated with high level of β-catenin (rank>7 folds; P = 0.009)(Table 1). However, there was no significant association between DVL3 expression and other clinical parameters, such as tumor stages (*P* = 0.369), grading (*P* = 0.658), metastasis (*P* = 0.243), β-catenin (*P* = 0.009) and CyclinD1 (*P* = 0.009). On the other hand, the up-regulation of β-catenin (rank >7 folds) was significantly associated with high grade tumors (*P* = 0.049) and over-expression of CyclinD1 (*P* = 0.009) but there was no significant correlation with tumor stages (*P* = 0.189) and metastasis (*P* = 0.345) (Table 2). Taken together, these findings indicate that the increased expression of DVL3 is correlated with β-catenin which is involved in cervical cancer development.

**Table 1:** Clinicopathological correlation of DVL3 expression in cervical cancer tissue array (CX1021) (Pantomics, CA, USA).

Characteristics	Total	DVL3 expression (Fold change)		
		≤5 folds	>5 folds	*P*
All cases	96	35 (36.5%)	61 (63.5%)	
Stage				
Early	64	21 (32.8%)	43 (67.2%)	
Late	32	14 (43.8%)	18 (56.2%)	0.369
Grade				
Low	54	21 (38.9%)	33 (61.1%)	
High	37	12 (32.4%)	25 (67.6%)	0.658
Metastasis				
No	70	23 (32.9%)	47 (67.1%)	
Yes	26	12 (46.2%)	14 (53.8%)	0.243
β-catenin				
≤7 folds	60	28 (46.7%)	32 (53.5%)	
>7 folds	36	7 (19.4%)	29 (80.6%)	0.009*
CyclinD1				
≤2 folds	38	20 (52.6%)	18 (47.4%)	
>2 folds	58	15 (25.9%)	43 (74.1%)	0.009*

**Table 2:** Clinicopathological correlation of β-catenin expression in cervical cancer tissue array (CX1021) (Pantomics, CA, USA).

Characteristics	Total	β-catenin expression (Fold change)		
		≤7 folds	>7 folds	*P*
All cases	96	60 (62.5%)	36 (37.5%)	
Stage				
Early	64	43 (67.2%)	21 (32.8%)	
Late	32	17 (32.8%)	15 (46.9%)	0.189
Grade				
Low	54	38 (70.4%)	16 (29.6%)	
High	37	18 (48.6%)	19 (51.4%)	0.049*
Metastasis				
No	70	46 (65.7%)	14 (53.8%)	
Yes	26	14 (53.8%)	12 (46.2%)	0.345
DVL3				
≤5 folds	35	28 (80%)	7 (20%)	
>5 folds	61	32 (52.5%)	29 (47.5%)	0.009*
CyclinD1				
≤2 folds	38	30 (78.9%)	8 (21.1%)	
>2 folds	58	30 (51.7%)	28 (48.3%)	0.009*

### DVL3 enhances proliferation of cervical cancer cells

**Figure 2:**
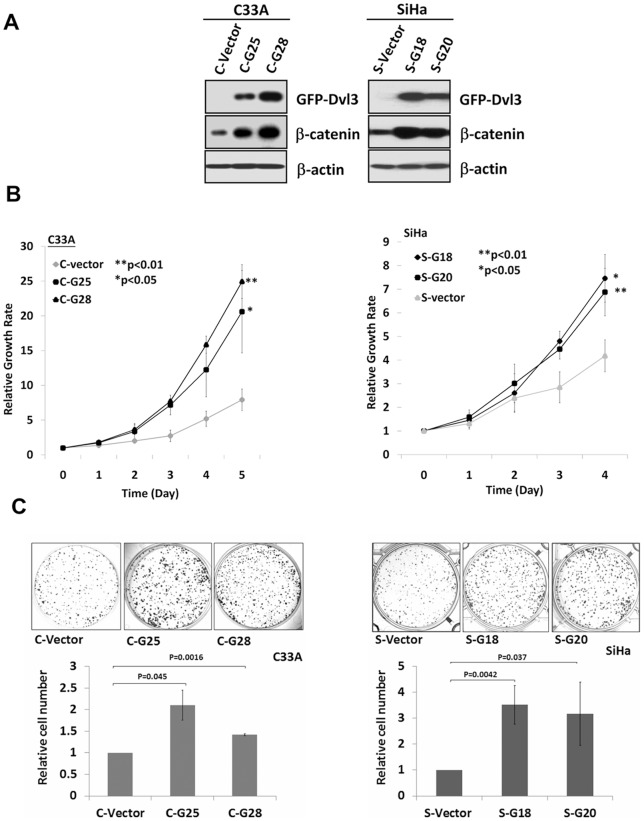
Ectopic expression of DVL3 enhances Wnt/β-catenin signaling activity and cell proliferation in cervical cancer cells. (A) Western blot showed that stably over-expressing GFP-DVL3 elevated expression of β-catenin in C33A (C-G25 and C-G28) and SiHa (S-G18 and S-G20). (B) XTT assay revealed a significant increase of cell proliferation in cervical cancer cells with stable expression of GFP-DVL3 (C-G24 and C-G28; *P*<0.05) (S-G18 and S-G20; *P*<0.05). (C) Clonogenic assay showed that twice and thrice the number of colonies were found in GFP-DVL3 ectopically expressing C33A cells (C-G25 and C-G28) (*P*<0.05) and SiHa cells (S-G18 and S-G20) (*P*<0.05) as compared with their vector controls.

DVLs are important signal transduction molecules mediating Wnt/β-catenin signaling activity to control cell growth. To address whether DVL3 could promote Wnt/β-catenin signaling activity, the dual luciferase reporter assay was performed. Transfection of DVL3-expressing plasmid into HEK293 cells increased β-catenin transcriptional activity in a dose-dependent manner (Figure 1D), suggesting that DVL3 is a positive regulator of the Wnt/β-catenin signaling cascade. Next, to investigate whether DVL3 promotes cell proliferation via activation of Wnt/β-catenin pathway in cervical cancer, two cervical cancer cell lines (C33A and SiHa) with stably expression of DVL3 were established using human GFP-tagged DVL3 expressing plasmid. Western blot analysis revealed that β-catenin was significantly elevated in GFP-DVL3 stable clones in C33A (C-G25 and C-G28) and SiHa (S-G18 and S-G20) (Figure 2A). Functionally, XTT cell proliferation assay revealed that ectopic expression of DVL3 significantly enhanced cell proliferation in GFP-DVL3 stable clones of SiHa (*P*<0.05) and C33A (*P*<0.001) cells (Figure 2B). In addition, clonogenic assay revealed that there were 2-3 fold increase in the number of colonies in SiHa cells stably expressing GFP-DVL3 (S-G18 and S-G20) (*P*<0.05) and C33A cells (C-G25 and C-G28) (*P*<0.05) respectively as compared with their vector controls (Figure 2C). Collectively, these findings suggest that DVL3 enhances Wnt/β-catenin signaling activity and promotes the proliferation of cervical cancer cells.

### AMPK activators reduce DVL3 in cervical cancer cells

**Figure 3:**
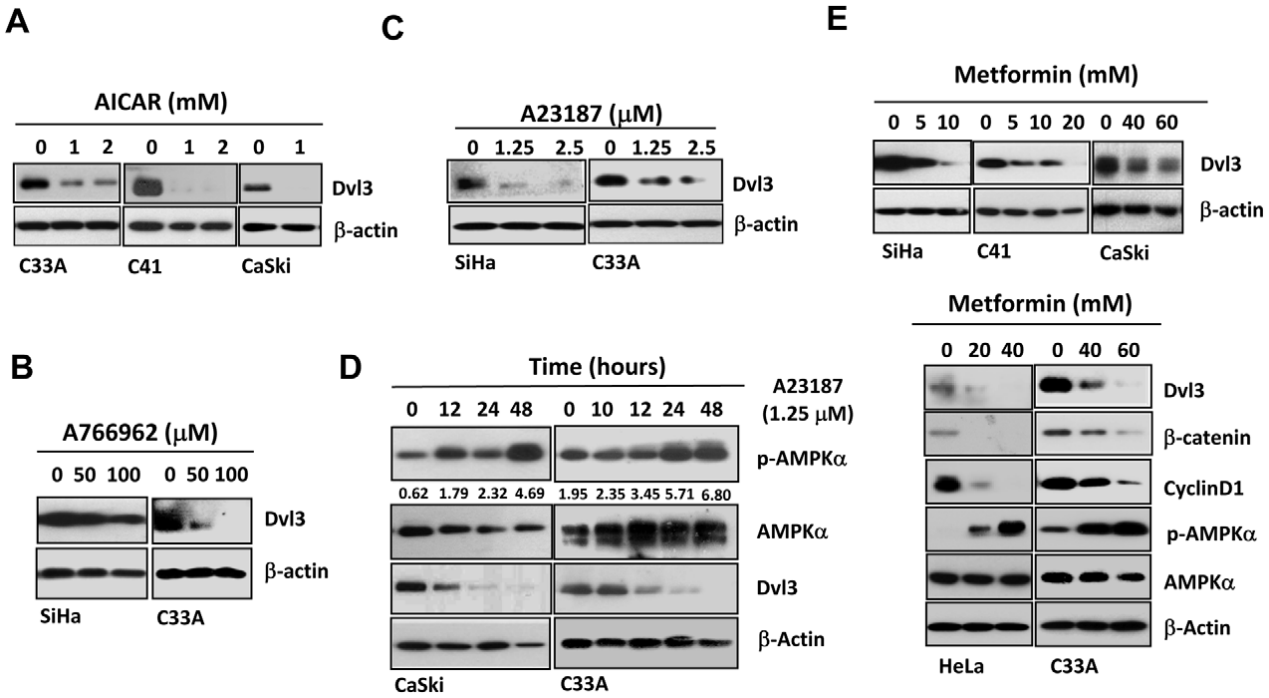
AMPK activators reduce DVL3 in cervical cancer cells. (A) Upon treatment of AICAR, approximately 80% reduction of DVL3 level was observed among C33A, C41 and CaSki. (B) Upon treatment of A769662 at different doses (0, 50 and 100 μM), a 20-30% and 80%–100% reduction of DVL3 in SiHa and C33A could be observed respectively. (C) Upon treatment with A23187 at different doses (0, 1.25 and 2.5 μM) for twenty hours, C33A and SiHa cells showed 80–90% reduction of DVL3 expression in a dose-dependent manner. (D) Upon treatment of 1.25 μM A23187, the decrease of DVL3 could be observed after 10 and 12 hours in C33A and CaSki respectively, while a further 100% reduction of DVL3 was observed at 12 and 24 hours for both cell lines. (E) Upon treatment of metformin, 60–80%reduction of DVL3 was observed in Hela, SiHa, CaSki, C41 and C33A in a dose-dependent manner.

**Figure 4:**
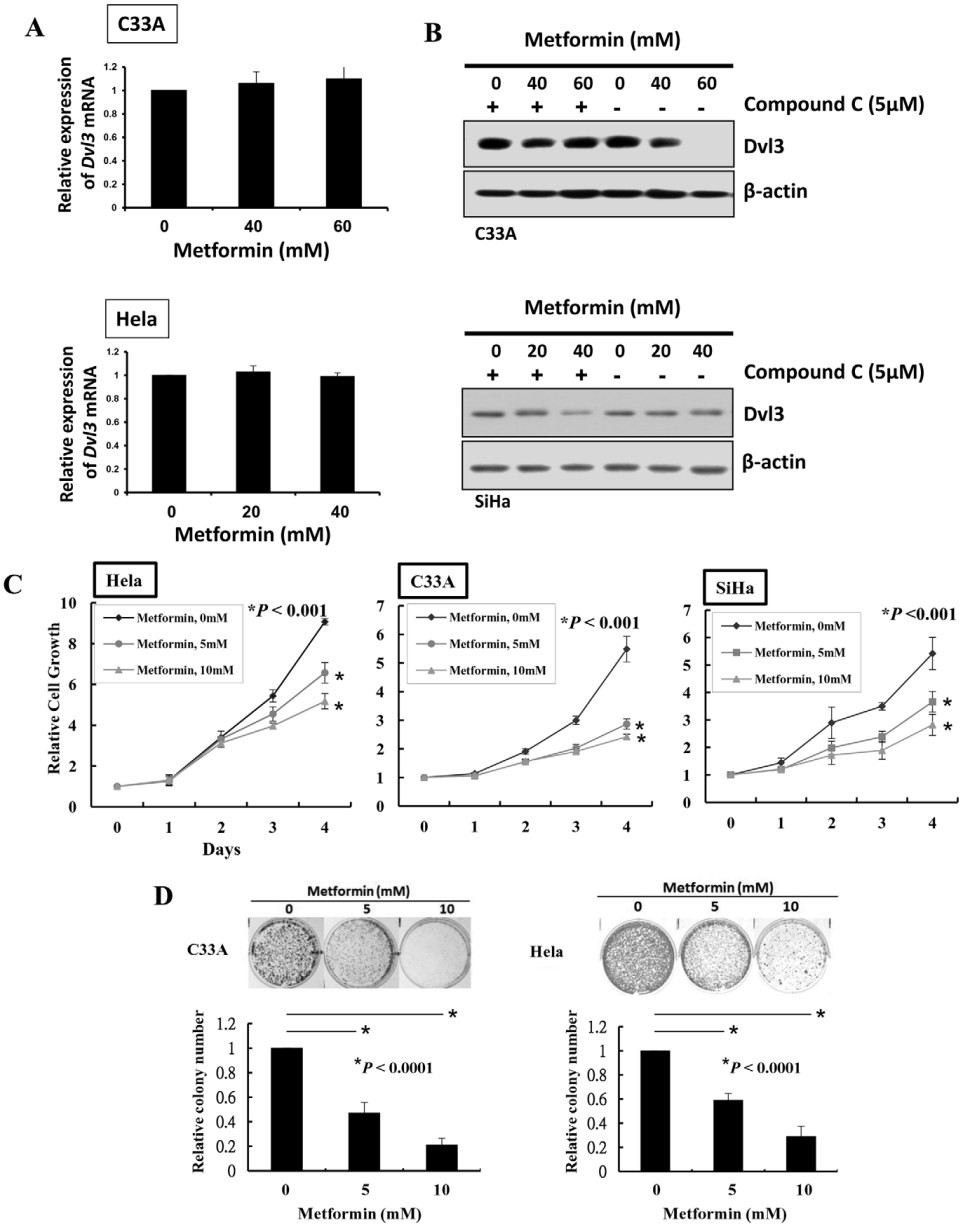
AMPK activation impairs cervical cancer cell growth through the reduction of DVL3. (A) Real time q-PCR analysis demonstrated that metformin had no effect on the expression of *DVL3* mRNA expression in C33A and HeLa cells. (B) Western blot analysis revealed that compound C could abate the decrement of DVL3 mediated by metformin. (C) XTT cell proliferation assay showed a significant reduction of cell proliferation in C33A (*P*>0.001), SiHa (*P*>0.001) and HeLa cells (*P*>0.001) treated with metformin in a dose-dependent manner. (D) Clonogenic assay showed a remarkable reduction of the number of colonies formed in HeLa and C33A treated with metformin in a dose-dependent manner.

Numerous studies have demonstrated that AMPK activators can inhibit cell growth in cancers, particularly in cervical cancer *in vitro* [21], [33], [34]. Therefore, we examined the effect of AMPK activators on the expression of DVL3 and its related Wnt/β-catenin signaling activity in cervical cancer cells. AICAR treatment resulted in significant reduction of DVL3 levels in three cervical cancer cell lines (Figure 3A). Another AMPK activator, A23187, activates AMPK through its upstream kinase (CaMKK) by increasing the cytosolic calcium level [20], also reduced DVL3 expression in C33A and SiHa cells in a dose-dependent manner (Figure 3C). In order to further assess the effect of A23187 on DVL3 expression, C33A and CaSki were treated with 1.25 μM A23187 and harvested at different time-points. Decrement of DVL3 was observed after 10 and 12 hours in C33A and CaSki respectively and no DVL3 was observed after 12 and 24 hours for both cell lines (Figure 3D). To further assess whether the reduction of DVL3 mediated by AMPK activators is a universal process, two well-known AMPK activators, A769662 and metformin, were used. A769662 is a thienopyridone which activates AMPK through interacting with the α- and β-subunit of AMPK. Upon A769662 treatment, DVL3 expression was abolished in a dose-dependent manner in SiHa and C33A (Figure 3B). In addition, metformin is a very common AMPK activator as well as a potential anti-cancer drug [22]. Upon treatment of metformin, several cervical cancer cell lines displayed differential responses to metformin and showed depletion of DVL3 in a dose-dependent manner (Figure 3E), along with no change in the mRNA level of *DVL3* in C33A and HeLa (Figure 4A), suggesting that the reduction of DVL3 regulated by AMPK activators was associated with post-transcriptional events. Taken together, these data imply that AMPK activators can reduce DVL3 and such effect is a universal process.

### AMPK activation is crucial for the reduction of DVL3 mediated by AMPK activators

Although the major role of AMPK activators is to activate AMPK activity, numerous reports have evidenced that some AMPK activators may exert off-target effects [35], [36]. Therefore, it is of interest to examine whether AMPK activators mediated reduction of DVL3 is solely dependent on AMPK signaling activity. We used a potent AMPK inhibitor, compound C, to counteract the effect of metformin on the AMPK activation. Consistently, reduction of DVL3 was observed after treatment of metformin in C33A and SiHa (Figure 4B), yet the decrement of DVL3 was abrogated with the addition of compound C. (Figure 4B). These data suggest that the reduction of DVL3 mediated by metformin is mainly through AMPK signaling activity.

### AMPK activators impair cervical cancer cell proliferation through reduction of DVL3 and Wnt/β-signaling activity

We have demonstrated that ectopic expression of DVL3 could enhance cell proliferation in cervical cancer cells. Therefore, to gain further insight into the function of depleted DVL3 in minimizing Wnt/β-catenin signaling and cell proliferation mediated by AMPK activators, C33A, SiHa and HeLa cells were treated with metformin and their proliferation rates were examined by XTT assay. Our results demonstrated that metformin significantly repressed cell proliferation in C33A (*P*>0.001), SiHa (*P*>0.001) and HeLa cells (*P*>0.001) (Figure 4C). Furthermore, clonogenic assay also revealed that the number of colonies was reduced by 20 and 50% in C33A and HeLa cells respectively (Figure 4D). These findings manifest that the inhibition of cervical cancer cell growth by AMPK activators is attributed to the reduction of DVL3.

### AMPK/mTOR activity is essential for the depletion of DVL3

**Figure 5:**
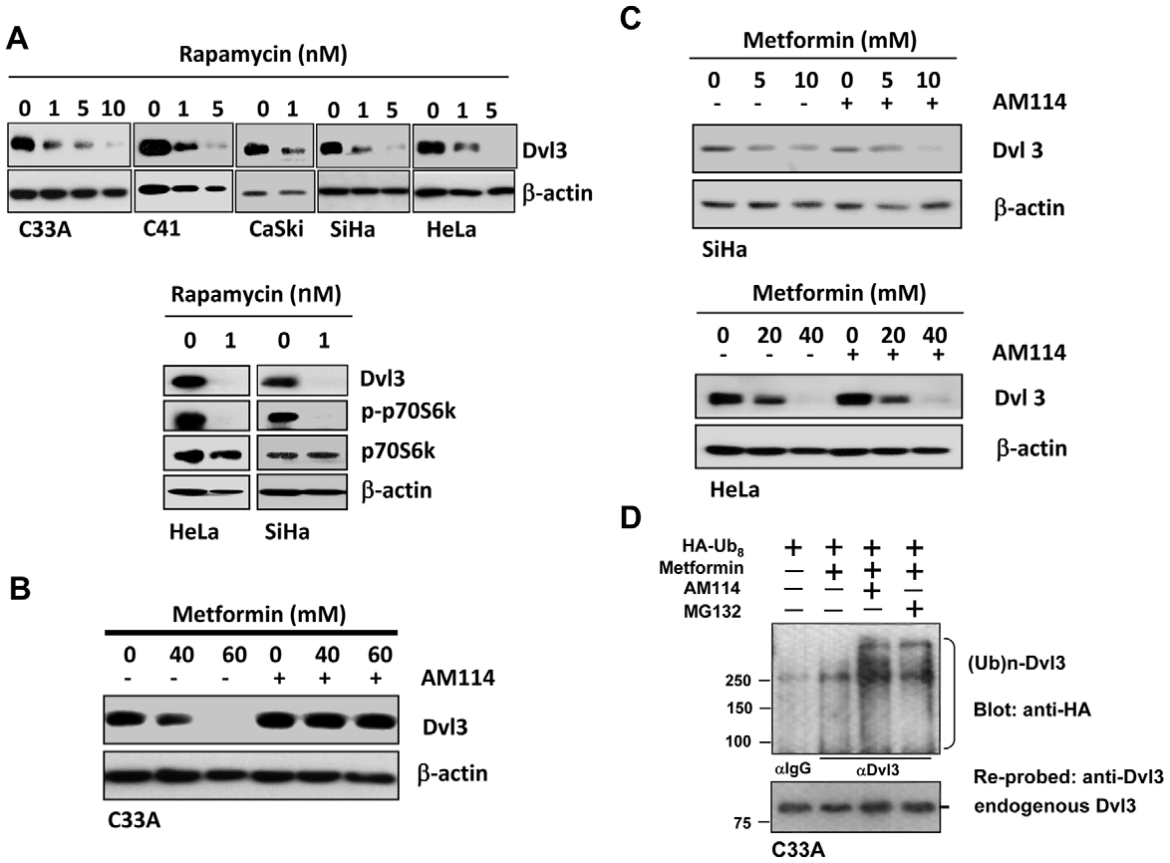
AMPK activators reduce DVL3 through either inactivation of mTOR activity or increased proteasomal degradation in cervical cancer cells. (A) Rapamycin inactivated p70S6 kinase activity and aborted protein synthesis of DVL3 in all cervical cancer cell lines (C33A, C41, CaSki, HeLa and SiHa) in a dose-dependent manner. (B) Proteasomal inhibitor, AM114, restored the reduced DVL3 mediated by metformin in C33A. (C) Proteasomal inhibitor, AM114, could not restore the reduced DVL3 mediated by metformin in SiHa and HeLa. (D) *In vivo* ubiquitination assay showed that DVL3 was degraded as poly-ubiquitinated DVL3 protein ((Ub)n-DVL3) in C33A cell model. C33A cells were transfected with pcDNA-HA(Ub)_8_ plasmid. Both MG132 or AM114 and metformin were used to enhance the amount of polyubiquitinated DVL3 protein products ((Ub)n-DVL3). Cell lysates were incubated with anti-DVL3, and the immunoprecipitates were probed with anti-HA. The same membrane was re-probed with anti-DVL3 to detect the expression of DVL3.

AMPK activators are able to activate AMPK and modulate its downstream cascade, such as the translational control of protein synthesis through inhibition of mTOR pathway [12], [13], [14]. Therefore, we reasoned the blockage of AMPK/mTOR activity can mimic the effects of AMPK activators on the decrement of DVL3. A mTOR inhibitor (Rapamycin) was employed to inhibit mTOR activity. Rapamycin suppresses the kinase activities of mTOR to inactivate p70S6k, thereby abolishing certain protein translation [37]. Upon treatment of Rapamycin, DVL3 was reduced in cervical cancer cell lines (C33A, C41, CaSki, HeLa and SiHa) in a dose-dependent manner. Furthermore, the inactivation of p70S6k in concomitant with the reduction of DVL3 was observed in HeLa and SiHa cells treated with 1 nM Rapamycin (Figure 5A). Taken together, the reduction of protein synthesis could reduce DVL3 expression in cervical cancer cell lines and that the decrement of DVL3 mediated by AMPK activators is mainly through the AMPK/mTOR signaling cascade.

### AMPK activators also promote proteasomal degradation of DVL3

Several lines of evidence have shown that DVLs are tightly regulated by proteasomal degradation mechanisms in which loss of factors involved in the ubiquitination pathways leads to DVLs accumulation [28], [38]. Thus, we reasoned that proteasomal degradation might serve as an alternative mechanism attributed to AMPK activators mediated DVL3 reduction. We used AM114, a proteasomal inhibitor, to suppress ubiquitin/proteasomal degradation. Upon co-treatment with AM114 and metformin, restored DVL3 expression was observed in C33A but not in SiHa and HeLa cells (Figure 5B and 5C). The *in vivo* ubiquitination assay further demonstrated that DVL3 in C33A cells could be degraded through ubiquitin/proteasome pathway because the formation of DVL3-ubiquitinated products was observed upon treatment of metformin and such DVL3-ubiquitinated products were further enhanced when co-treatment with either AM114 or MG132 (Figure 5D). These data indicate that the proteasomal degradation also plays a role in the reduction of DVL3 mediated by AMPK activators.

## Discussion

In this study, we have shown that DVL3 was aberrantly over-expressed and significantly correlated with increased β-catenin in cervical cancer. Our data also demonstrated that DVL3 could promote cervical cancer growth through activating Wnt/β-catenin signaling pathway. More importantly, we addressed the potential use of AMPK activators in reducing DVL3 and hence inhibiting the cervical cancer cell growth. Besides, this is the first report showing the possible molecular mechanisms of how AMPK activators impair cervical cancer cell growth through the crosstalk between AMPK and the Wnt/β-catenin signaling.

Aberrant activation of Wnt/β-catenin signaling pathway has been documented in various human cancers including cervical cancer [39]. Indeed, mounting evidences have shown that DVLs which are positive regulators of the Wnt/β-catenin signaling pathway are frequently upregulated in a variety of human cancers [32], [40], [41], [42], [43], [44], [45], [46]. The overexpressed DVLs are closely associated with the increased Wnt/β-catenin activity in human cancers [31], [32], [47]. However, very little is known about the functional roles of DVLs in cervical cancer. Herein, we observed that DVL3, but neither DVL1 nor DVL2, was significantly up-regulated and associated with augmented Wnt/β-catenin signaling activity and cervical cancer cell growth.

Numerous studies have reported that the upstream kinases of AMPK, like LKB1, is frequently mutated and deleted in lung, breast and especially cervical cancers [21], [48], [49], thereby lowering AMPK activities for cancer cell growth. Indeed, low AMPK activity is a common event favoring cancer cell growth [15], [16]. Thus, AMPK is a crucial target in cancer therapy. We and other groups have previously demonstrated that several AMPK activators could suppress cancer cell growth [21], [22], [50]. However, the molecular mechanisms of the repression remain largely unexplored. Previously, Kohno's group showed that metformin reduced the protein level of β-catenin through regulating its phosphorylation-dependent degradation [51]. This gave us a clue that metformin may regulate upstream regulators of the Wnt/β-catenin signaling cascade. Herein, we demonstrated that remarkable reduction of DVL3 could be induced by multiple AMPK activators, suggesting that AMPK activators universally reduce DVL3. In line with previous reports on the anti-tumor effect of metformin through down-regulating the CyclinD1 level in prostate cancer and breast cancer [52], [53], the reduction of DVL3 expression is also in concomitant with the decreased levels of β-catenin and its downstream target, CyclinD1, which is responsible for cell cycle control. Surprisingly, metformin only affects the protein level of DVL3 but not its mRNA, indicating that the regulation of DVL3 mediated by AMPK activators relies on post-transcriptional modification.

As multiple AMPK activators could reduce DVL3, we speculated that AMPK activity is essential in such regulation. In order to test whether AMPK activators exert their effects on DVL3 through the activation of AMPK, an AMPK inhibitor, compound C, was co-treated with metformin in cervical cancer cells to counteract the effect of AMPK activation. As expected, compound C abated the function of metformin in attenuating DVL3 expression. These findings manifest that AMPK activators could reduce DVL3 through modulating AMPK activity, and such effect is not due to AMPK activators' off-targeted functions. Indeed, *in vitro* cell proliferation assays also revealed that the use of AMPK activators (e.g. metformin) could reduce cell proliferation. We provide solid evidence that AMPK activators, especially metformin, exert a down-regulating effect on DVL3 and hence reduce the expression levels of β-catenin and CyclinD1, and ultimately attenuate cell proliferation. More importantly, we are the first to show that AMPK activators exert their anti-proliferative effect through reducing DVL3 and Wnt/β-catenin signaling activity. AMPK activation plays a requisite role in suppressing tumor development in cervical cancer. These findings underscore the therapeutic value of targeting DVL3 using AMPK activators in cervical cancer treatment.

Activation of AMPK activity suppresses mTOR signaling through inhibiting p70S6 kinase activity and thereby attenuates protein translation [37], [54], [55]. We therefore reasoned that the suppression of mTOR signaling could affect the translational efficiency of DVL3. In fact, treatment of an mTOR inhibitor, Rapamycin, could abolish DVL3 in cervical cancer cell lines. Also, Rapamycin could lower the expression level of β-catenin, suggesting that Rapamycin exhibited similar behaviors as AMPK activators in the reduction of Wnt/β-catenin signaling through regulating DVL3 expression [56]. On the other hand, we may also question that the suppression of protein synthesis mediated by mTOR/p70S6 inhibition may reduce other important cellular proteins simultaneously. However, the high proliferative capacity of cervical cancer cells closely relies on Wnt/β-catenin signaling activity and its associated oncoprotein levels [10], [57]. Therefore, DVL3, a key oncoprotein in activating Wnt/β-catenin signaling, should have a higher impact on cell growth upon the treatment of AMPK activators in cervical cancer cells.

Aforementioned, numerous reports have suggested that proteasomal degradation plays important role in regulating DVLs. For examples, DVLs are ubiquitinated by different ubiquitin ligases, such as NEDL1 and KLHL12-Cullin-3 [58], [59], or factors involved in the ubiquitination-degradation such as Prickle-1, Dapper1, myristoylated Naked2 and Down syndrome critical region protein (Dscr5) [28], [38], [60], [61]. Therefore, we reasoned that proteasomal degradation was involved in the reduction of DVL3 induced by AMPK activators. As expected, AMPK activators mediated down-regulation of DVL3 could be restored after suppression of proteasomal degradation by AM114 or MG132. However, such effect was only observed in C33A but not in SiHa, indicating that proteasomal degradation mechanism only plays a minor role in the regulation of DVL3 by AMPK activators in cervical cancer cells.

In conclusion, we revealed the oncogenic role of DVL3 in augmented Wnt/β-catenin signaling activity in cervical cancer. More importantly, AMPK activators could suppress over-expressed DVL3 mainly through attenuation of AMPK/mTOR mediated protein synthesis and partially regulated by promoting ubiquitin/proteasome mechanism. Altogether, these findings underscored the value of AMPK activators as promising anti-cancer agents in cervical cancer therapy. The understanding of the regulation of AMPK activators on DVL3 will assist in the exploration of alternative anti-cancer drugs for clinical usage.
